# Mindfulness Mechanisms in Sports: Mediating Effects of Rumination and Emotion Regulation on Sport-Specific Coping

**DOI:** 10.1007/s12671-017-0711-4

**Published:** 2017-05-03

**Authors:** Torbjörn Josefsson, Andreas Ivarsson, Magnus Lindwall, Henrik Gustafsson, Andreas Stenling, Jan Böröy, Emil Mattsson, Jakob Carnebratt, Simon Sevholt, Emil Falkevik

**Affiliations:** 1 Center of Research on Welfare, Health, and Sport, Halmstad University, Kristian IV:s väg 3, 301 18 Halmstad, Sweden; 2 Department of Food and Nutrition, and Sport Science; Department of Psychology, University of Gothenburg, Läroverksgatan 5, 405 30 Gothenburg, Sweden; 3 Faculty of Health, Science, and Technology, Karlstad University, Universitetsgatan 2, 651 88 Karlstad, Sweden; 4 Department of Psychology, Umeå University, 901 87 Umeå, Sweden

**Keywords:** Coping, Emotion regulation, Mindfulness, Performance, Rumination, Sport

## Abstract

The main objective of the project was to examine a proposed theoretical model of mindfulness mechanisms in sports. We conducted two studies (the first study using a cross-sectional design and the second a longitudinal design) to investigate if rumination and emotion regulation mediate the relation between dispositional mindfulness and sport-specific coping. Two hundred and forty-two young elite athletes, drawn from various sports, were recruited for the cross-sectional study. For the longitudinal study, 65 elite athletes were recruited. All analyses were performed using Bayesian statistics. The path analyses showed credible indirect effects of dispositional mindfulness on coping via rumination and emotion regulation in both the cross-sectional study and the longitudinal study. Additionally, the results in both studies showed credible direct effects of dispositional mindfulness on rumination and emotion regulation. Further, credible direct effects of emotion regulation as well as rumination on coping were also found in both studies. Our findings support the theoretical model, indicating that rumination and emotion regulation function as essential mechanisms in the relation between dispositional mindfulness and sport-specific coping skills. Increased dispositional mindfulness in competitive athletes (i.e. by practicing mindfulness) may lead to reductions in rumination, as well as an improved capacity to regulate negative emotions. By doing so, athletes may improve their sport-related coping skills, and thereby enhance athletic performance.

## Introduction

In sport psychology, competitive athletes are taught psychological strategies to better cope with a number of demanding challenges related to psychological functioning (Birrer et al. 2012). Even if the majority of successful athletes usually would be considered as psychologically healthy, they may still experience a wide range of internal processes such as competition anxiety, negative emotions, fear of failure and dysfunctional thinking that may influence performance negatively (Birrer et al. 2012). In addition, personality-related factors, for instance avoidant coping styles, as well as interpersonal problems may also inhibit performance (Birrer et al. 2012).

Traditionally, psychological skills training (PST), based on cognitive-behavioural principles, has been applied to develop increased self-control over internal processes (e.g. mental and emotional) that may inhibit performance (Moore 2009). However, during the last decade, several researchers have pointed out that the empirical support for regular PST, in relation to athletic performance, is limited (Moore 2009).

Drawing from contemporary clinical research (i.e. Hayes et al. 1999), Gardner and Moore (2004) introduced a mindfulness- and acceptance-based programme, specifically designed for athletic performance enhancement, as an alternative to PST. Mindfulness is usually described as a certain kind of present-centred non-judgemental awareness of internal and external stimuli where an individual attends to all these events on a moment-to-moment basis without trying to control, change or avoid any of these internal experiences (e.g. Brown et al. 2007; Kabat-Zinn 1994).

In a sport context, Gardner (2009) states that mindfulness may not directly cause an effect on sport performance; the effect is rather hypothesized to go indirectly through another variable that results in improved athletic performance. This hypothesis is supported by Röthlin et al. (2016) who found that competition anxiety mediated the relationship between dispositional mindfulness and self-rated sport performance.

In an attempt to clarify what mindfulness is and what its mechanisms are, Coffey et al. (2010) factor analysed several mindfulness-related self-report scales and finally came up with a two-component solution, consisting of present-centred attention and acceptance of experiences. Moreover, the results indicated that rumination and negative emotion regulation may serve as important mechanisms explaining mindfulness-related health outcomes (Coffey et al. 2010). Rumination is characterized by highly self-focused and repetitive, “unstoppable”, negative thoughts. Increased rumination is associated with psychological distress, depression, worry and anxiety (Nolen-Hoeksema 2000). Moreover, reductions in rumination have been found to mediate the relations between dispositional mindfulness and reductions in psychological distress as well as increased well-being (Coffey et al. 2010; Jain et al. 2007).

Emotion regulation refers to a capacity to manage negative and/or challenging emotions and has been defined as “the processes by which individuals influence which emotions they have, when they have them, and how they experience and express these emotions” (Gross 1998, p. 275). Moreover, emotion regulation should be regarded as an ability to manage and adaptively respond to negative emotions, rather than a process where distressing emotions are controlled, inhibited or eliminated (Gratz and Roemer 2004). In accordance with this view, adaptive emotion regulation has been conceptualized as a multidimensional construct involving the following: “(a) awareness, understanding, and acceptance of emotions; (b) ability to engage in goal-directed behaviors, and inhibit impulsive behaviors, when experiencing negative emotions; (c) flexible use of situationally-appropriate strategies to modulate the intensity and/or duration of emotional responses, rather than to eliminate emotions entirely; and (d) willingness to experience negative emotions as part of pursuing meaningful activities in life” (Gratz and Tull 2010, p. 111). The above conceptualization of emotion regulation overlaps with aspects of the mindfulness construct (Roemer et al. 2015). For instance, a frequently used conceptualization of mindfulness includes non-reactivity to inner experiences (Baer et al. 2006), in some aspects very similar to one of the above core features of emotion regulation. In addition, both emotion regulation and mindfulness strongly emphasize acceptance of emotions (Roemer et al. 2015). Numerous studies support the idea that mindfulness-based interventions are related to an improved ability to regulate negative emotions in clinical populations (see Roemer et al. 2015, for an overview). Coffey et al. (2010) discovered that emotion regulation, similar to rumination, was a mediator in the relation between dispositional mindfulness and psychological distress and well-being. An increased mindful awareness may in itself change how people relate to internal experiences, such as thoughts and emotions, by a proposed meta-mechanism, reperceiving, reflecting a shift from a self-centred perspective to an objective perspective (Shapiro et al. 2006). An improved ability to relate objectively to events may prevent people from getting mentally and emotionally caught up in experiences (Shapiro et al. 2006) that may make it easier for individuals to quickly detect negative emotions that need to be regulated (Roemer et al. 2015). Reperceiving is believed to enhance emotional as well as cognitive flexibility that, in turn, may lead to increased affect tolerance and reductions in emotional intensity, negative evaluations of emotions, worry and rumination (Roemer et al. 2015; Shapiro et al. 2006).

Another theoretical view is taken by Grabovac et al. (2011), who introduced the Buddhist psychological model (BPM). The theory states that decreased mental proliferation is the main mechanism, explaining psychological health outcomes due to mindfulness practice. Mental proliferation is described as “habitual reactions of attachment and aversion to the pleasant, unpleasant, and neutral feelings of prior sense impressions and mental events” (Grabovac et al. 2011, p. 157). Increased mental proliferation will most likely result in rumination (Grabovac et al. 2011). Thus, reductions in rumination may play a crucial part in explaining how mindfulness practice influences mental health. However, neither emotion regulation nor rumination has specifically been empirically examined as potential mindfulness-related mechanisms in athlete populations.

In an ambitious effort to increase the understanding of the role mindfulness has in sport, Birrer et al. (2012) set out to develop a working model that specifically tries to explain how and why mindfulness may enhance athletic performance. Birrer and Morgan (2010) argued that athletes need to cultivate several psychological skills (e.g. motivation skills, coping skills, attention skills and recovery skills) that may help them to cope with various sport-specific requirements (e.g. complex movement patterns, strenuous training scope and injury). Psychological techniques used in a sport context, such as goal setting and imagery, are applied for the purpose of promoting and strengthening psychological skills that may facilitate peak athletic performance (Birrer and Morgan 2010).

Dispositional mindfulness, reflecting a trait-like ability to be mindful in everyday life, needs to be differentiated from mindfulness practice (Brown et al. 2007). Birrer et al. (2012) proposed that the concept of mindfulness practice, primarily based on conceptualizations suggested by Shapiro et al. (2006) and Dorjee (2010), may consist of three facets: (i) intention to practice; (ii) bare attention—a Buddhist term, defined as “the clear and single-minded awareness of what actually happens to us, and in us, at the successive moments of perception” (Thera 1996, p. 30); and (iii) attitude (acceptance, openness, self-respect and non-reactivity). Regarding the operationalization of dispositional mindfulness, Birrer et al. (2012) used the four-factor model, developed by Bergomi et al. (2013): (i) accepting, nonreactive and insightful orientation, (ii) present awareness, (iii) describing of experiences and (iv) open, non-avoidant orientation.

Because mindfulness is seen as a complex, multi-component concept, Birrer et al. (2012) suggest that mindfulness may, indirectly, through a number of certain impact mechanisms, influence several psychological sport-related skills. Drawing on contemporary mindfulness research (e.g. Coffey et al. 2010; Dorjee 2010; Shapiro et al. 2006), Birrer et al. (2012) developed a theoretical model on how aspects of mindfulness practice as well as dispositional mindfulness components may lead to nine specific impact mechanisms (bare attention, attitude, values clarification, self-regulation/negative emotion regulation, clarity, exposure, flexibility, non-attachment and less rumination). These mechanisms are hypothesized to improve 11 domains of psychological skills that may favour athletic performance (personal development and life skills, self-skills, recovery skills, coping skills, motivation skills, pain management skills, attentional skills, arousal regulation skills, perceptual-cognitive skills, motor control skills and communication and leadership skills). The impact mechanism less rumination is hypothesized to enhance several skills, among them, arousal regulation and coping. Similarly, negative emotion regulation is suggested to improve skills such as coping and self-skills (Birrer et al. 2012).

By identifying specific mindfulness mechanisms and how they might be related to psychological skills and performance enhancement, this working model (Birrer et al. 2012) is, indeed, a good starting point in trying to understand how mindfulness works, and the model will certainly be useful in empirical examinations of mindfulness mechanisms in sports as well as in other performance-related domains. However, there are concerns about apparent overlaps between aspects of mindfulness (mindfulness practice and dispositional mindfulness) and features of some impact mechanisms.

First, the mindfulness construct has repeatedly been associated with attitudinal qualities such as acceptance and openness in theoretical models (e.g. Coffey et al. 2010; Shapiro et al. 2006), and Birrer et al. (2012) conceptualizations of both dispositional mindfulness and mindfulness practice thereby include attitudinal qualities. However, because attitude is also categorized as a proposed impact mechanism, it will be difficult to statistically analyse a model in which a core feature of the independent variable to a large extent is identical with its potential mechanism.

Second, attention is widely regarded as a core component in the mindfulness concept (e.g. Coffey et al. 2010; Shapiro et al. 2006) and has subsequently also been included as a prominent dimension in many current self-report measures designed to assess trait-mindfulness (see Bergomi et al. 2013, for an overview). Attention is regarded as both an impact mechanism and a psychological skill (Birrer et al. 2012). Thus, it will be methodologically very challenging to operationalize three different aspects of attention: as a feature of the mindfulness concept (independent variable), as a certain mechanism (mediator/mechanism) and finally, as an outcome (dependent variable). It will be statistically nearly impossible to investigate if mindfulness (including an attention component) leads to an attention impact mechanism that subsequently will result in improved attentional skills.

## Study 1

**Fig. 1:**
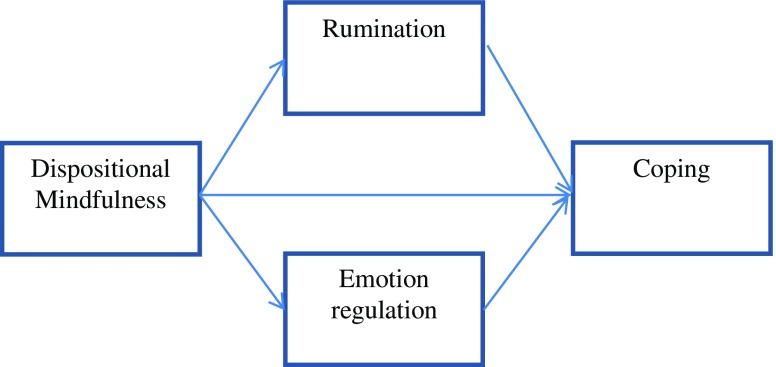
Hypothesized model

In line with Birrer et al. (2012) recommendations, the current research project aims at exploring the relations between dispositional mindfulness, impact mechanisms and psychological skills. Taking other theories of mindfulness mechanisms into account (Coffey et al. 2010; Grabovac et al. 2011) as well as the aforementioned problems of overlaps between mindfulness and mechanisms in the Birrer et al. (2012) model, we finally chose to include two of what we regarded as major potential mechanisms: emotion regulation and rumination. Sport-specific coping was chosen as the dependent variable because coping successfully with sport-related demands is associated with improved performance (Nicholls et al. 2016). Using a cross-sectional design, the aim of study 1 was to examine if rumination and emotion regulation could mediate the relationship between dispositional mindfulness and sport-specific coping. For an illustration of the hypothesized model, see Fig. 1. For the purpose of studying the temporal relationships between mindfulness, mechanisms and skills, as proposed in the Birrer et al. (2012) model, a longitudinal design was employed in study 2, including three time points.

## Method

### Participants

Two hundred and forty-two young competitive elite athletes (172 men and 69 women, mean age = 18. 4, *SD* = 0.26) were recruited from 12 sports associations and two sport high schools in the southwest area of Sweden. The participants were drawn from a variety of six sports: football (soccer) (*n* = 134), handball (*n* = 21), ice hockey (*n* = 38), athletics (*n* = 8), equestrian sport (*n* = 5) and floorball (*n* = 36). The elite levels among the participants were as follows: local (*n* = 10), regional (*n* = 73), national (*n* = 124) and international (*n* = 30).

### Procedure

Letters including a presentation of the current study and an inquiry to let their athletes participate were sent by email to 16 sports associations in southwest Sweden of which 12 agreed to take part in the study. Additionally, two sport high schools were invited to participate (both agreed to participate). The data were collected in November 2014 and were conducted at the place of each sports association and sport high school. The APA ethical standards were followed in the conduct of the study. All participants were given written and verbal information about the study. They were further informed that the data would be treated confidentially and that they had the right to quit the study at any time. Written informed consent was obtained from all participants included in the study, prior to the first data collection. Participants did not receive any compensation for taking part in the study.

### Measures

#### The Five-Facet Mindfulness Questionnaire

Four of the five subscales in the Swedish short-form 29-item version of the Five-Facet Mindfulness Questionnaire (FFMQ) (Lilja et al. 2011), designed to measure mindfulness skills in daily life, were used to assess dispositional mindfulness (non-reactivity to inner experience, observing, acting with awareness and non-judging of experience). Responses were given on a 5-point Likert scale, ranging from 1 (never or very rarely true) to 5 (very often or always true). The subscale describe was excluded because several researchers argue that it is theoretically weakly linked to the mindfulness construct (e.g. Josefsson and Broberg 2010). High scores indicate a high level of dispositional mindfulness. The original version of the FFMQ has shown good psychometric properties (Baer et al. 2008), and the Swedish version has shown similar results: Cronbach’s alpha coefficients for the five subscales ranged from 0.75 to 0.85 (Lilja et al. 2011). In the cross-sectional study, Cronbach’s alpha for the total scale (in which describe was excluded) was 0.56.

#### Difficulties in Emotion Regulation Scale

Four of the six subscales in the Difficulties in Emotion Regulation Scale (DERS; Gratz and Roemer 2004) were combined into a total emotion regulation scale (difficulties engaging in goal directed behaviour, impulse control difficulties, lack of emotional awareness, limited access to emotion regulation strategies). The DERS-subscale non-acceptance of emotional responses was excluded because it was considered to be too similar to the FFMQ-subscale nonjudging of experience. The close relationship between these two subscales was also confirmed in factor analyses in Coffey et al. (2010) where the scales together comprised one factor. Moreover, the subscale clarity was also excluded because it has been suggested to be a distinct mechanism in itself, separated from emotion regulation, theoretically (Birrer et al. 2012) as well as in factor analyses (Coffey et al. 2010). The complete DERS is a 36-item questionnaire designed to assess multiple aspects of emotional dysregulation, using a 5-point Likert scale ranging from 1 (almost always) to 5 (almost never). Higher scores indicate greater problems with emotion regulation. Validity as well as reliability has in general been shown to be consistent across various populations (Ritschel et al. 2015). The remaining four DERS-subscales were thus computed into a total emotion regulation scale (Cronbach’s alpha = 0.80).

#### Rumination Reflection Questionnaire

The rumination subscale in the Rumination Reflection Questionnaire (RRQ; Trapnell and Campbell 1999) was used to assess the proposed impact mechanism rumination. The subscale contains 12 items on a 5-point Likert scale (1 = strongly disagree to 5 = strongly agree). Cronbach’s alpha of the original subscale was 0.90 (Trapnell and Campbell 1999). Cronbach’s alpha in the current study was 0.82.

#### Athletic Coping Skills Inventory

In order to assess coping skills, the Athletic Coping Skills Inventory (ACSI; Smith et al. 1995) was used. ACSI is a 28-item scale and consists of seven subscales (coping with adversity, coachability, concentration, confidence and achievement motivation, goal setting and mental preparation, peaking under pressure, freedom from worry), using a 4-point Likert scale (0 = almost never to 3 = almost always). In the present study, only the total scale was used in the analyses (Cronbach’s alpha = 0.87), which is similar for the total scale in the original version (Cronbach’s alpha = 0.86; Smith et al. 1995).

### Data Analyses

All analyses were performed using Bayesian statistics. The foundation of Bayesian estimation is to “reallocate belief toward the parameter values that are consistent with the data and away from parameter values that are inconsistent with the data” (Kruschke 2013, p. 574). One of the major differences between the frequentist and the Bayesian paradigm is that all unknown parameters, within the Bayesian paradigm, can incorporate (un)certainty that can be defined by a probability distribution (for an extended discussion about the differences between frequentist and Bayesian statistics, see Ivarsson et al. 2015; Stenling et al. 2015; Zyphur and Oswald 2015). Specifically, it is the credibility of parameters that best adapt to the observed data that is warranted. In contrast, the same parameters, within the frequentist paradigm, are fixed but unknown (Van de Schoot and Depaoli 2014). To reallocate the credibility, the Bayes’ rules equation is used (Bayes and Price 1763). The formula will generate the probability (posterior) of the parameter values given the data by multiplying the probability of the data given the parameter values (likelihood) and the prior probabilities of parameter values (prior) (Zyphur and Oswald 2015). There are different algorithms for doing the data generation, but the most common is the Markov chain Monte Carlo (MCMC; Asparouhov and Muthén 2010). In the MCMC, a great number of combined parameter values are generated that represent credible parameter values. These credible parameter values reconcile with the prior distribution combined with the observed data. The credible parameter values will generate a representation of the posterior distribution that is used to interpret the posterior probability of, for example, regression coefficient (Kruschke 2013).

Descriptive statistics. Demographic characteristics and background variables were analysed using the JASP software package (Love et al. 2015). Bayesian correlation analyses were conducted to investigate the relationships between dispositional mindfulness, rumination, emotion regulation, coping, age and level of participation. For all analyses, a Bayes factor (BF) was calculated. The BF quantifies the evidence, provided by the observed data, of one statistical hypothesis over the other (H_0_ vs H_A_). More specifically, a BF larger than 1 indicates stronger evidence for H_A_ in comparison to H_0_ (Ivarsson et al. 2015). In the present study, BF above 10 was determined to be evidential (Andraszewicz et al. 2015). A BF of 10 indicates, for example, that H_A_ is ten times more likely, in the observed data, than H_0_.

#### Path Analysis

Also, for the path analysis, the Bayesian estimator was used. To test for mediation effects, we used the approach, suggested by Yuan and MacKinnon (2009) that “allows the user to determine the posterior distribution of the indirect effect αβ, together with a 95% credible interval” (Nuijten et al. 2015, p. 87). In study 1, we used the default non-informative prior distribution in Mplus 7.4 (Muthén 2010). Model convergence was assessed with the potential scale reduction factor (PSRF; Brooks and Gelman 1998), and a PSRF around 1 is considered as evidence of convergence (Kaplan and Depaoli 2012). We implemented Bayesian models using MCMC simulation procedures with a Gibbs sampler and specified a fixed number of 300,000 iterations for each of the four MCMC chains. Model convergence was assessed using statistical criteria (i.e. PSRF <1.1; see Asparouhov and Muthén 2010) and visual inspection of trace plots to ensure that multiple chains converged to a similar target distribution (Van de Schoot et al. 2014).

Model fit of the BSEM models was assessed using the posterior predictive *p* (PP*p*) value and the 95% credibility interval. A well-fitting model should have a PP*p* value around 0.50 in combination with a symmetric 95% credibility interval centering on zero.

For each parameter, a credibility interval was calculated. In contrast to the frequentist confidence interval, the credibility interval allows the researcher to calculate an interval that indicates the probability (e.g. 95%) that the parameter of interest lies between the two values given the observed data. This is an intuitive and meaningful interpretation that is easier to communicate than the frequentist confidence interval because it provides the probability that a certain parameter lies between two numbers (Van de Schoot et al. 2014). If the 95% credibility interval did not include zero, we used the recommendations from Zyphur and Oswald (2015) and concluded that the null hypothesis was rejected as improbable.

## Results

The results from the correlation analyses showed strong evidence (BF > 10) for positive relationships between emotion regulation and rumination (*r*s = .23 to .41). Dispositional mindfulness had negative relationships with emotion regulation (*r* = −.41) and rumination (*r* = −.48), but a positive relationship with coping skills (*r* = .44). Coping was negatively correlated with emotion regulation and rumination (*r*s = −.24 to −.40). Age was negatively related to emotion regulation (*r* = −.26). For more information, see Table 1.

**Table 1:** Descriptive statistics and correlations between the variables in study 1 and study 2

Variable	Study 1 M (SD)	Study 2 M (SD)	1	2	3	4	5
1. Mindfulness	3.17 (0.33)	3.16 (0.35)	1	−.41*	−.48*	.44*	.15
2. Rumination	3.06 (0.64)	2.97 (0.77)	−.39	1	.41*	−.36*	−.07
3. Emotion regulation	2.65 (0.47)	2.46 (0.48)	−.42*	.43*	1	−.40*	−.26*
4. Coping	2.86 (0.35)	2.86 (0.31)	.20	−.42	−.36	1	.16
5. Age	18.42 (4.04)	22.78 (4.66)	.10	−.25	−.30	.25	1

Note: Correlations between study variables for study 1 appear above the diagonal. Correlations between study variable for study 2 appear below the diagonal. In study 2, mindfulness was measured at T1, rumination and emotion regulation were measured a T2 and coping was measured at T3

*BF > 10

The path model indicated a good data-model fit (PP*p* = .499, 95% CI [−13.96, 14.24]). Dispositional mindfulness had negative direct effects on rumination and emotion regulation. Also, dispositional mindfulness had a positive direct effect on coping skills. Emotion regulation and rumination both had negative direct effects on coping skills. Dispositional mindfulness accounted for 16% of the variance in rumination and 21% of the variance in emotion regulation. Also, these variables accounted for 27% of the variance in coping skills. For a summary of all direct effects, see Table 2.

**Table 2:** Summary of standardized direct effects tested in study 1 and 2

	Β [95% credibility interval]	
	Study 1	Study 2
Mindfulness → rumination	−.41 [−.50, −.29]	−.36 [−.47, −.26]
Mindfulness → ER	−.46 [−.55, −.35]	−.45 [−.55, −.34]
Mindfulness → coping	.26 [.13, .38]	.03 [−.24, .29]
ER → coping	−.23 [−.36, −.10]	−.27 [−.41, −.13]
Rumination → coping	−.15 [−.28, −.02]	−.21 [−.36, −.08]

Note: In study 2, mindfulness was measured at T1; rumination, emotion regulation and clarity were measured at T2; and coping was measured at T3

*ER* emotion regulation

In the hypothesized model, two indirect pathways were included. The result showed both of the indirect effects to be credible (i.e. the credibility interval did not include zero). Dispositional mindfulness had an indirect effect on coping skills via emotion regulation (αβ = 0.11, 95% CI [0.05, 0.19]). Also, dispositional mindfulness had an indirect effect on coping skills via rumination (αβ = 0.06, 95% CI [0.01, 0.13]).

## Study 2

The aim of study 2 was to examine if rumination and emotion regulation (all measured at T2) mediate the relationship between dispositional mindfulness (measured at T1) and psychological skills (i.e. coping skills, measured at T3), using a longitudinal design. For an illustration of the hypothesized model, see Fig. 1.

## Method

### Participants

Sixty-five competitive elite athletes (32 men and 33 women, mean age = 22.8, *SD* = 4.66) were recruited from sports associations and sport high schools in the southwest area of Sweden. The participants were drawn from two sports: football (soccer) (*n* = 54) and athletics (*n* = 11). The competitive level among the participants ranged from regional (*n* = 1) to national (*n* = 52), to international (*n* = 12).

### Procedure

Letters including a presentation of the current study and an inquiry to let their athletes participate in a repeated measure study were sent by email to sports associations as well as sport high schools in southwest Sweden. The data collections were conducted at the place of each sports association and sport high school. The data were collected at three separate times during a period of 4 weeks in March and April 2015. Thus, the interval was approximately 2 weeks between each data collection. The APA ethical standards were followed in the conduct of the study. All participants were given written and verbal information about the study. They were further informed that the data would be treated confidentially and that they had the right to quit the study at any time. Written informed consent was obtained from all participants included in the study, prior to the first data collection. Participants did not receive any compensation for taking part in the study.

### Measures

The same questionnaires as in study 1 were used in study 2. Cronbach’s alpha estimates for measures in study 2 were as follows: FFMQ-total scale (four subscales): 0.65 (T1), DERS-total scale (four subscales): 0.89 (T2), rumination: 0.91 (T2) and ACSI-total scale: 0.81 (T3).

### Data Analyses

For the analyses of the data in study 2, we used the same approach as we did in study 1. Priors for the structural parameter estimates were obtained from the empirical findings in study 1. Because different priors potentially can influence the relation between variables (Zyphur and Oswald 2015), a sensitivity analysis was performed. In the sensitivity analysis, the hypothesized model (i.e. using empirical priors) was compared with two other models, using the same mean parameter but with different variance priors. In one of the comparison models, a highly precise prior was used for the variance whereas, in the second comparison model, we used priors with low precision (see Table 3).

**Table 3:** Comparison of unstandardized parameter estimates of using different priors

	Prior mean (variance)	Model A	Model B	Model C
M → R	−.79 (.014)	−.81 [−1.02, −.60]	−.79 [−.85, −.73]	−.85 [−.1.33, −.37]
M → ER	−.69 (.008)	−.67 [−.82, −.51]	−.69 [−.75, −.63]	−.60 [−.93, −.27]
ER → C	−.17 (.002)	−.17 [−.25, −.09]	−.17 [−.23, −.11]	−.15 [−.40, .11]
R → C	−.08 (.001)	−.09 [−.15, −.03]	−.09 [−.15, −.03]	−.14 [−.30, .03]
IND R	NA	.07 [.03;.13]	.07 [.03, .12]	.12 [−.02, .30]
IND ER	NA	.11 [.06; .18]	.12 [.07, .16]	.09 [−.06, .28]

Note: Model A = hypothesized model with empirical priors for parameter estimates and variances; model B = highly precise priors were set for the expected parameter estimates variances (i.e. .001); model C = low precise priors were set for the expected parameter estimates variances (i.e. .02). Mindfulness was measured at T1; rumination, emotion regulation and clarity were measured at T2; and coping was measured at T3

*M* dispositional mindfulness, *R* rumination, *ER* emotion regulation, *C* coping, *IND R* indirect effect between dispositional mindfulness and coping via rumination, *IND ER* indirect effect between dispositional mindfulness and coping via emotion regulation

## Results

The results from the correlation analyses showed strong evidence (BF > 10) for positive relationships between emotion regulation and rumination (*r* = .43). Also, dispositional mindfulness had a negative relationships with emotion regulation (*r* = −.42). The complete correlation matrix is displayed in Table 1.

All three models demonstrated a good data-model fit (PP*p* values ranged between .476 and .584). Also, the parameter estimates did not differ substantially between the different models. Therefore, we will focus our results on the hypothesized model with empirical priors for parameter estimates and variances (see Table 3).

The path model indicated a good data-model fit (PP*p* = .574, 95% CI [−15.19, 12.21]). Dispositional mindfulness at T1 had negative direct effects on rumination and emotion regulation at T2. There were also credible direct effects of emotion regulation and rumination at T2 and on coping skills at T3. Dispositional mindfulness at T1 accounted for 20% of the variance in emotion regulation and 13% in rumination at T2. The three variables accounted for 22% of the variance in coping at T3. For a summary of all direct effects, see Table 2.

In the hypothesized model, two indirect effects were estimated. The result showed credible indirect effects between dispositional mindfulness at T1 and coping skills at T3 via emotion regulation (αβ = 0.11, 95% CI [0.06, 0.18]) and rumination (αβ = 0.07, 95% CI [0.03, 0.13]), both measured at T2. For more information, see Table 3.

## Discussion

We conducted two separate studies (the first study using a cross-sectional design and the second a longitudinal design) to investigate if rumination and emotion regulation mediate the relation between dispositional mindfulness and sport-specific coping in an athlete population. In line with the proposed theoretical model of Birrer et al. (2012), and also consistent with previous empirical research (Coffey et al. 2010; Röthlin et al. 2016), a credible indirect effect of dispositional mindfulness on coping via rumination and emotion regulation was found in both studies. The path analyses also showed credible direct effects of dispositional mindfulness on rumination and emotion regulation. Further, credible direct effects, in the expected direction, of emotion regulation and rumination on coping were found in both studies. Hence, our findings support the theoretical model, suggested by Birrer et al. (2012), indicating that rumination as well as emotion regulation may be essential mechanisms in the relation between dispositional mindfulness and sport-specific coping skills.

Athletes who have a trait-like ability to be mindful in daily life tend to regulate their negative emotions effectively and not engage in excessive rumination, which may, in turn, improve their coping skills in relation to a variety of sport-related challenges. Dispositional mindfulness may increase the ability for athletes to be aware of and understand potential performance-inhibiting emotions and thoughts. Further, dispositional mindfulness may also make it easier for the competitive athlete to “cool down” the intensity of arousal and strong emotions in general, and also to shorten the duration of their presence. In line with the theory proposed by Shapiro et al. (2006), a mindful athlete may have the capacity to “reperceive” (objectively relate to experiences). Reperceiving, in turn, is hypothesized to improve self-management, increase cognitive and emotional flexibility as well as affect tolerance, and thereby preventing the athlete from being too caught up in distressing emotions and negative thoughts when facing various sport-related challenges. Moreover, mindfulness practice and its associated increase in dispositional mindfulness may lead to a greater objective awareness of internal as well as external stimuli.

Borkovec (2002) argued that a present-centred, externally oriented mind optimally processes information, in which less attention is paid to internal operations, possibly resulting in less ruminative and/or self-evaluative thoughts. A mindful mind that is not preoccupied with self-centred thoughts may be better equipped to regulate distressing emotions in comparison with a mindless mind. In contrast, a mindless, self-focused mind may easily get stuck in thought cycles and their accompanying emotions. An athlete who has a relatively quiet, non-ruminative mind, and who can regulate negative emotions, may be able to focus exclusively on goal-directed behaviours, such as current task-relevant stimuli. By doing so, the athlete may be optimally prepared to make the right decision in the present moment and adaptively cope with current challenges. This may be especially important for athletes when facing crucial moments in competitions, for example when a competition does not go as well as planned or when something unexpected happens during a competition. A process such as the one described above may arguably create the ideal conditions for peak athletic performance. Hence, to regulate negative emotions effectively and to not engage in rumination appear to be important mechanisms for adaptive coping in a sport context. The slightly larger effect estimates for emotion regulation may suggest that healthy emotion regulation may be of particular importance for athletes’ perceived coping skills.

In sum, increased dispositional mindfulness in competitive athletes (e.g. by practicing mindfulness) may lead to reductions in rumination, as well as an improved capacity to regulate negative emotions. By doing so, athletes may improve their sport-related coping skills, and thereby enhance athletic performance. Furthermore, our findings also provide plausible and testable hypotheses of why sport-related mindfulness-based interventions have shown statistically significant effects on sport performance in previous studies (e.g. Bernier et al. 2009; John et al. 2011). However, there are several other potentially important mindfulness mechanisms in the Birrer et al. (2012) model, aside from rumination and emotion regulation, that also may be related to psychological sport-skills and athletic performance.

## Limitations

Several methodological limitations need to be recognized in the present study. First, due to the limitations of a cross-sectional design in study 1, we are unable to make causal inferences. For this reason, alternative models can be equally possible, for example, less emotion regulation difficulties leading to increases in dispositional mindfulness and coping. Similarly, the results in the longitudinal study are also compatible with alternative explanations, like enhanced coping skills facilitating improved emotion regulation, which in turn enhances dispositional mindfulness. Nevertheless, because our aim was to investigate the model developed by Birrer et al. (2012), the proposed direction of effects was followed.

Second, the sample size in study 2 was quite small, making the generalizability to the target population rather limited. This small sample might influence the accuracy of the estimates. Also, the specified priors will have larger impact on results in models with small samples.

Third, the temporal intervals between the data collections in study 2 are relatively short, only 2 weeks. Moreover, it has been highlighted that longitudinal research should be based on a theoretical model of how and when within-changes occur over time as well as the shape of change (Stenling et al. 2017). Even if our longitudinal design is based on a theoretical model (Birrer et al. 2012), the theoretical assumptions do not, however, specify when these proposed changes occur. Further, the shape of change is not explicitly described in the model but a plausible interpretation is that changes in variables are assumed to occur linearly. Additionally, the expected time intervals between the independent variable, the mediator and the dependent variables have not been clarified in the model. In other words, the Birrer et al. (2012) model does not specify when changes in emotion regulation and rumination due to enhanced mindfulness occur, and further, when these changes are expected to result in improved coping skills. In general, the mindfulness literature does not reveal much about how quickly changes in dispositional mindfulness occur or to what extent the process of change occurs periodically or continuously, and further, when changes in dispositional mindfulness lead to changes in other variables. However, a few studies investigating the effects of short-term mindfulness-based interventions (4- to 6-week programmes) have shown significant pre- to post-test increases in dispositional mindfulness and psychological well-being (e.g. Josefsson et al. 2014). Furthermore, positive and statistically significant effects on emotion regulation and anxiety after only a single brief mindfulness session have been reported (e.g. Erisman and Roemer 2010; Feldner et al. 2003). These findings indicate that mindfulness-related changes may occur rather rapidly, suggesting that a time interval of 2 weeks in the present longitudinal study may not necessarily be too short for changes to arise.

Fourth, a limitation in the cross-sectional study is that Cronbach’s alpha estimate of the FFMQ total scale was rather low. In general, low alpha estimates are related to random errors, and results involving less reliable scales need to be cautiously interpreted (Mitchell and Jolley 2012). In this case, it may mean that the items in the total FFMQ-scale measure different aspects of mindfulness that do not completely capture the same phenomenon.

Finally, the aforementioned overlaps between conceptualizations of mindfulness and emotion regulation may to some extent explain the strong estimates found in the current studies. In an attempt to deal with this overlapping problem, we have, similar to Coffey et al. (2010), excluded subscales in the DERS that were considered to be too much alike certain subscales in the FFMQ. Still, the two constructs do appear to partly reflect a similar phenomenon, addressing the need to further distinguish the constructs from each other (Gratz and Tull 2010).

Future research should examine mediating effects of rumination and emotion regulation between mindfulness and psychological skills in a longitudinal mindfulness-based sport-specific intervention study. In addition, we would suggest that sport performance is measured, and also that dispositional mindfulness is assessed using a sport-related mindfulness measure, such as the recently developed Athletic Mindfulness Questionnaire (AMQ; Zhang et al. 2015), in which mindfulness has been operationalized as a multidimensional concept consisting of present-moment attention, awareness and acceptance.
